# Supplementary material for: Sustained-input switches for transcription factors and microRNAs are central building blocks of eukaryotic gene circuits
Source: Genome Biol. 2013 Aug 23;14(8):R85. doi: 10.1186/gb-2013-14-8-r85 (PMC4054853; doi:10.1186/gb-2013-14-8-r85)
Supplement: Additional file 5 — HTML Browsable Motif Output. Zipped folder containing all WaRSwap and FANMOD motif output, viewable in a web browser. [file gb-2013-14-8-r85-S5.ZIP › HTML_browsable_motif_output/WaRSwap_all_tair9/sigs_WSWRSLDraw-2000_all.pvals.heatmaps.html/motif_id_46_000101110_tftype_all_upstream_-2000_0.html]

```
BG_MODEL = Rnew2
MOTIF_ID = 46_000101110
TF_TYPE = all
UPSTREAM = -2000_0


PVals
FN_0.2	FN_0.4	FN_0.6	FN_0.8
dg_60.genes	0.041	0.001	0.09	0.571
dg_70.genes	0.036	0.002	0.104	0.545
dg_80.genes	0.041	0.002	0.098	0.562

ZScores
FN_0.2	FN_0.4	FN_0.6	FN_0.8
dg_60.genes	1.744	2.699	1.302	-0.325
dg_70.genes	1.803	2.658	1.298	-0.269
dg_80.genes	1.747	2.647	1.302	-0.322

StDevs
FN_0.2	FN_0.4	FN_0.6	FN_0.8
dg_60.genes	2467.476	758.062	178.29	11.385
dg_70.genes	2392.617	760.242	179.583	11.023
dg_80.genes	2454.669	757.659	173.185	11.494
```
